# Study protocol of the Bergen brain-gut-microbiota-axis study: A prospective case-report characterization and dietary intervention study to evaluate the effects of microbiota alterations on cognition and anatomical and functional brain connectivity in patients with irritable bowel syndrome

**DOI:** 10.1097/MD.0000000000021950

**Published:** 2020-09-11

**Authors:** Birgitte Berentsen, Bharath Halandur Nagaraja, Erica Pearson Teige, Gülen Arslan Lied, Astri J. Lundervold, Katarina Lundervold, Elisabeth Kjelsvik Steinsvik, Eline Randulff Hillestad, Jørgen Valeur, Ingeborg Brønstad, Odd Helge Gilja, Berge Osnes, Jan Gunnar Hatlebakk, Judit Haász, Jennifer Labus, Arpana Gupta, Emeran A. Mayer, Alfonso Benitez-Páez, Yolanda Sanz, Arvid Lundervold, Trygve Hausken

**Affiliations:** a National Center for Functional Gastrointestinal Disorders, Haukeland University Hospital, Bergen, Norway; b National Center for Ultrasound in Gastroenterology, Medical Department, Haukeland University Hospital, Bergen, Norway; c Center for Nutrition, Department of Clinical Medicine, University of Bergen, Bergen, Norway; d Mohn Medical Imaging and Visualization Center, Haukeland University Hospital, Bergen, Norway; e Department of Biological and Medical Psychology, University of Bergen, Bergen, Norway; f Microbial Ecology, Nutrition & Health Research Unit, Institute of Agrochemistry and Food Technology, National Research Council (IATA-CSIC), Valencia, Spain; g Department of Radiology, Haukeland University Hospital, Bergen, Norway; h G. Oppenheimer Center for Neurobiology of Stress and Resilience, UCLA Vatche and Tamar Manoukian Division of Digestive Diseases, and UCLA Microbiome Center, David Geffen School of Medicine at UCLA, Los Angeles, CA; i Unger-Vetlesen Institute, Lovisenberg Diaconal Hospital, Oslo, Norway.

**Keywords:** brain-gut-microbiota axis, functional magnetic resonance imaging, irritable bowel syndrome, machine learning, neurogastroenterology, psychometric tests, transabdominal ultrasound

## Abstract

**Introduction:**

Irritable bowel syndrome (IBS) is a common clinical label for medically unexplained gastrointestinal (GI) symptoms, recently described as a disturbance of the brain-gut-microbiota (BGM) axis. To gain a better understanding of the mechanisms underlying the poorly understood etiology of IBS, we have designed a multifaceted study that aim to stratify the complex interaction and dysfunction between the brain, the gut, and the microbiota in patients with IBS.

**Methods:**

Deep phenotyping data from patients with IBS (n = 100) and healthy age- (between 18 and 65) and gender-matched controls (n = 40) will be collected between May 2019 and December 2021. Psychometric tests, questionnaires, human biological tissue/samples (blood, faeces, saliva, and GI biopsies from antrum, duodenum, and sigmoid colon), assessment of gastric accommodation and emptying using transabdominal ultrasound, vagal activity, and functional and structural magnetic resonance imaging (MRI) of the brain, are included in the investigation of each participant. A subgroup of 60 patients with IBS-D will be further included in a 12-week low FODMAP dietary intervention-study to determine short and long-term effects of diet on GI symptoms, microbiota composition and functions, molecular GI signatures, cognitive, emotional and social functions, and structural and functional brain signatures. Deep machine learning, prediction tools, and big data analyses will be used for multivariate analyses allowing disease stratification and diagnostic biomarker detection.

**Discussion:**

To our knowledge, this is the first study to employ unsupervised machine learning techniques and incorporate systems-based interactions between the central and the peripheral components of the brain-gut-microbiota axis at the levels of the multiomics, microbiota profiles, and brain connectome of a cohort of 100 patients with IBS and matched controls; study long-term safety and efficacy of the low-FODMAP diet on changes in nutritional status, gut microbiota composition, and metabolites; and to investigate changes in the brain and gut connectome after 12 weeks strict low-FODMAP-diet in patients with IBS. However, there are also limitations to the study. As a restrictive diet, the low-FODMAP diet carries risks of nutritional inadequacy and may foster disordered eating patterns. Strict FODMAP restriction induces a potentially unfavourable gut microbiota, although the health effects are unknown.

**Trial registration number:**

NCT04296552 (ClinicalTrials.gov)

**Protocol version 1 May 2019**

## 1 Introduction

Irritable bowel syndrome (IBS) is a symptom-based diagnosis characterized by chronic abdominal pain associated with altered bowel habits, in the form of diarrhea, constipation, or a mix between the two.^[1]^ IBS is the most common condition encountered by gastroenterologists, with a global pooled prevalence of 11.2%^[2]^ and a prevalence around 20% in the western world.^[3]^ In the absence of a structural or organic cause, growing evidence suggests that there is a plethora of factors that may elicit abnormal responses in patients with IBS. Collectively, these factors are described as dysregulation of the brain-gut-microbiota (BGM) interaction. As a bidirectional link between the brain and the gut, the axis reciprocally interacts with key sensorimotor functions, sympathetic and parasympathetic branches of the autonomic nervous system, the endocrine and immune system, the hypothalamic-pituitary-adrenal-axis, the enteric nervous system (ENS), and gut microbiota and metabolites. Alterations in gut microbiota composition or functions is known to impact human behaviour and brain physiology, and dysbiosis is commonly reported in patients with IBS.^[4,5]^ However, whether IBS-symptoms are caused by changes in microbiota still remains elusive and multiple pathways and mechanisms have been suggested to be involved. These include immune, endocrine, and neural signalling pathways, which the gut microbiota and metabolites may modulate. For instance, structural components of intestinal bacteria and bacterially produced metabolites [e.g., short chain fatty acids (SCFAs), tryptophan metabolites] may regulate immune function and cytokine production, with upstream effects on the blood–brain barrier or brain function through modulation of neuroinflammation. The gut microbiota, directly or through interactions with the host or diet, produce neuroactive compounds (gamma-aminobutyric acid, 5-hydroxytryptamine receptor, norepinephrine, etc) that may affect neuroendocrine functions locally or through humoral or neural routes such as signaling along the vagal nerve.^[6]^

Indeed, there is a substantial knowledge gap in regards to understanding pathophysiology in both the brain and the gut. The current view on disorders of the BGM axis has replaced the conventional focus on individual brain regions and cell types of the gut, integrating brain networks (brain connectome) and networks of gut cells and microbiota (gut connectome).^[7]^ In this study, we will employ cutting-edge omics and advanced computational methods to unravel gastrointestinal (GI) pathophysiology of IBS, link this information to results from multimodal brain imaging examination, gastric accommodation test, and information from psychometric tests and questionnaires, and translate these into clinical biomarkers. By this, we aim to stratify the diagnosis and improve treatment for patients with IBS.

## 2 Methods/Design

### 2.1 Objectives

The primary objective of this study is to increase knowledge and understanding of BGM axis dysfunction and identify covariance across multiple phenotypes leading to identification of biomarkers.

The secondary objectives are addressing the multifaceted manifestations of BGM axis dysfunction, specifically

- To identify and quantify structural and functional brain connectivity signatures, including visceral pain provocation and psychophysiological measures, using advanced neuroimaging techniques and statistical approaches.
- To investigate clinical biochemistry in blood (plasma, serum).
- To evaluate upper GI motility patterns using transabdominal ultrasound.
- To investigate GI biopsies for multi-omics analysis and complex molecular subclassification.
- To identify microbiota signatures and metabolites associated with gut-brain equilibrium or dysfunction in IBS patients and healthy controls.
- To investigate clinical effects of short and long-term strict low-FODMAP diet.
- To examine and compare the compositional and functional response of gut microbiota at baseline and after the low-FODMAP-dietary intervention, and the relationship with clinical and psychophysiological outcomes.
- To evaluate if the GA-map technology can be used to predict the response to treatment (to distinguish responder and non-responders prior to low-FODMAP diet).
- To investigate cognitive and emotional function associated with brain structures and functions known to be affected in adults with IBS, and personality traits and behaviour expected to be of importance to the patient's everyday functioning and effects of a strict low-FODMAP diet.
- To define phenotypes and biomarkers from all our abovementioned data using high dimensional data-driven and machine learning techniques.

### 2.2 Study population, recruitment, and consent

Eligible patients from the Bergen area are recruited from the IBS outpatient clinic at Haukeland University Hospital and through social media. Patients are informed about the study online (www.braingut.no.) and are individually informed by telephone by a study nurse, before inclusion. If a patient decides to participate in the study, (s)he signs an informed consent. Patient recruitment will take place between May 2019 and December 2021. Inclusion criteria for the studyincluded age between 18 and 65 years; fulfillment of the Rome-IV criteria for IBS: Reports of recurrent abdominal pain on average at least 1 day per week during the previous 3 months that is associated with alterations in bowel habits; duration at least 6 months and an IBS-symptom severity score (SSS) >175.^[8]^ Exclusion criteria for the study included pharmacological treatment affecting the GI-tract, including treatment for depression; presence of an organic disease such as coeliac disease, inflammatory bowel disease, diabetes, active *Helicobacter pylori* infection, polycystic ovary syndrome, and neurological diseases such as multiple sclerosis, Parkinson's disease, and amyotrophic lateral sclerosis; treated with systemic antibiotics within the last 3 months; probiotics or low-FODMAP-diet within the last 3 weeks or being vegan or vegetarian; regular use of analgesics; pregnancy; previous intestinal surgery except appendectomy; claustrophobia or having metallic implants that are not MRI compatible; been travelling outside Europe within the last 3 weeks (or plan to travel in the nearest future); participation in any other simultaneous clinical study; inability to comprehend and respond to questionnaires or follow dietary guidance.

Three patients and one healthy volunteer will be included in the study every 4 weeks until the necessary number of participants have been enrolled (IBS n = 100 patients, HC n = 40) at baseline. Sixty candidates with IBS-D will enroll in the 12-week strict low-FODMAP dietary intervention. Additional patients will be enrolled to replace patients who drop out during baseline until the end of the inclusion period. Patients who unexpectedly get claustrophobic during brain scanning may be included in the dietary intervention without functional magnetic resonance imaging (fMRI) examination.

### 2.3 Study design

The study is an open, single-center, case–control characterization study, followed by open label dietary intervention for a subgroup of subjects with IBS-D. Table 1 gives an overview of the study procedures and events during case–control characterization (baseline) and the following 12-week strict low-FODMAP diet intervention.

**Table 1:**
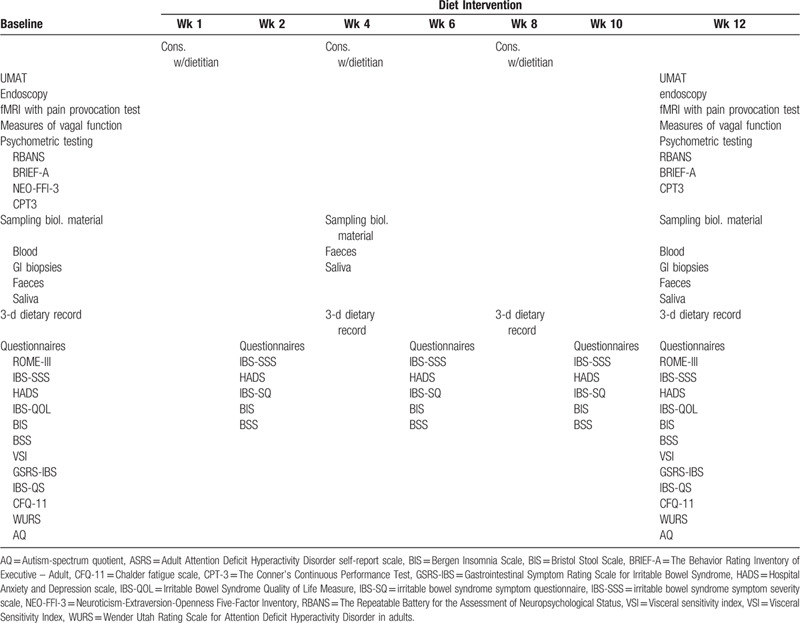
Brain-gut study procedures and events during baseline and 12-week strict low-FODMAP diet intervention.

## 3 Outcomes

### 3.1 Primary outcome

Primary outcomes included proportion of patients with treatment success in the lowFODMAP dietary intervention. Treatment success is defined as an improvement of ≥50 points on the IBS symptom severity score (IBS-SSS) at 12 weeks after treatment start compared with the score at baseline.

### 3.2 Secondary outcomes

- ≥50 points decrease in the IBS symptom severity score (IBS-SSS) from the scores 4 weeks after treatment compared to baseline.
- ≥13 points increase in the IBS-Qualiy of Life (IBS-QoL) week 4 and/or week 12 after treatment when compared to the scores at baseline
- Twenty percent reduction on visual analog scale on nausea, pain, and bloating measured post lactulose-test at week 12, compared with baseline scores.
- Proportion of patients with change dysbiosis index at week 4 and week 12 according GA-map technology, compared with baseline and/or healthy controls.

### 3.3 Explorative outcomes

- Changes in structural and functional brain connectivity signatures, including visceral pain provocation and psychophysiological measures at 12 weeks compared with baseline, and healthy controls.
- Changes in resting state regional brain interactions at 12 weeks compared with baseline and healthy controls, as measured with fMRI.
- Changes in cortical thickness (FA values) as measured with DTI (MRI)
- Differences in taxonomy and function of the microbiome, the immune system, metabolome and gut epithelial barrier in IBS patients at 12 weeks compared with baseline measures, and healthy controls.
- Changes in levels of SCFA composition, compared with baseline, and heathy controls.
- Changes in cognitive and emotional function, personality traits, and behavior at 12 weeks compared with baseline, and healthy controls.
- Improvements in The Conner's Continuous Performance Test (CPT-3) at 12 weeks in IBS patients, compared with baseline.

### 3.4 Sample size estimation

Generally, for samples size calculation and statistical power, we would make the following assumptions: Anticipated effect size (f^2^) is medium (0.20), desired statistical power level is 0.8 (80%) with type-I error of 5% (α = 0.05). On the basis of these assumptions, the minimum required samples size is 59 in each group for multiple regression analyses. Previous experience with this patient group has shown that we may expect a high level of missing data. To allow diagnostic subclassification, we therefore have increased the baseline sample size to n = 100, and the dietary intervention samples size n = 60 in patients with IBS-D and n = 40 for healthy controls. However, to identify biomarkers and patient subpopulations, comprehensive analysis methods will be employed to combine neuroimaging and neuromics data with clinical patient data. Although IBS is a highly heterogeneous patient group, we believe machine learning techniques and possibly pre-trained models will enable us to make inferences about an IBS population from the given sample, considering the limitation of cost, time, and convenience of data collection.

### 3.5 Clinical assessment

#### 3.5.1 Structural and functional brain MRI

Participants will be scanned using a Siemens Biograph mMR PET/MRI, incorporating a 3 T MRI Verio scanner with MQ gradients (45 mT/m @200 T/m/s), a 12-channel RF head coil and paralell imaging. The following MRI sequences will be applied: Scout (136 images, voxelsize 1.6 x 1.6 x 1.6 mm^3^; iPAT = 3; TA 0:14); T1-w 3D MPRAGE (192 slices, voxelsize 1.0 x 1.0 x 1.0 mm^3^; TR/TE/TI = 2400/2.26/900 ms, FA = 8 deg; iPAT = 2; TA_5:35), T2-w 3D TSE (192 slices; voxelsize 1.0 x 1.0 x 1.0 mm^3^; TR/TE = 3200/407 ms, FA = 120 deg; iPAT = 2; TA 5:12), GRE field map (36 slices, voxelsize 3.0 x 3.0 x 3.75 mm^3^; TR/TE1/TE2 = 400/4.92/7.38 ms, FA = 60 deg; iPAT = 1; TA 0:54); rs-fMRI epi2d moco (47 slices, voxelsize 2.4 x 2.4 x 3.12 mm^3^, 200 vols; TR/TE = 2890/30 ms, FA = 90 deg; iPAT = 2; TA 9:48), rs-fMRI epi2d moco (32 slices, voxelsize 2.4 x 2.4 x 3.12 mm^3^, 200 vols; TR/TE = 1980/30 ms, FA = 90 deg; iPAT = 2; TA 6:43), dMRI MMDW (47 slices, voxelsize 2.4 x 2.4 x 3.12 mm^3^;TR/TE = 8000/113 ms, FA = 90 deg; iPAT = 2; diff.scheme = bipolar; phase encoding A<<P; bvecs = 30 directions, bvals = 0 (n = 1)/1000 (n = 30)/2500 (n = 30) s/mm^2^; TA 8:34, 8 PA epi2d diff (47 slices, voxelsize 2.4 x 2.4 x 3.12 mm^3^; TR/TE = 7200/113 ms, FA = 90; iPAT = 2; phase encoding P>>A; bvals = 0; TA 0:30. Total examination time ∼45 min.

Functional MRI, using blood-oxygenation-level dependent (BOLD) contrast, will be used to record neuronal activity (i.e., neurovascular responses) in both IBS patients and healthy controls. Resting-state fMRI is a special “task-free” variant in which the subject is asked to lie as still as possible in the head coil while being continuously scanned, to investigate spontaneous brain activity (resting state networks).^[9–12]^ Diffusion imaging (dMRI) involves measuring of water diffusion in brain tissue. This provides information on a voxel-by-voxel basis about white matter microstructure, properties of with matter fiber bundles in the brain.^[13–15]^ For this study, we do not plan to use the opportunity of truly simultaneous acquisition of structural and functional information of the same brain regions from both MRI and PET. Our cerebral MRI protocol is a partly compliant with and motivated by the standardized Pain and Interoception Imaging Network protocol.^[16]^

#### 3.5.2 Psychometric testing

The Repeatable Battery for the Assessment of Neuropsychological Status (RBANS) is a brief, individually administered test-battery designed as a screening instrument to evaluate neuropsychological status of adults, ages 20 to 89 years.^[17]^ The test battery includes 12 subtests giving measures of attention, language, visuospatial/constructional abilities, and immediate and delayed memory function, in addition to a composite score and estimates of intellectual function according to available Scandinavian norms. In the present study, the results will be used for defining the cognitive function of the participants, as a covariate in the statistical analyses, and for establishing norms for patients with IBS.

The Behavior Rating Inventory of Executive Function (BRIEF-A) will be used to assess executive functions (EFs), as they are experienced in the daily life of the participants.^[18]^ The scale includes 86 items in 8 clinical scales (Inhibit, Shift, Emotional Control, Initiate, Working Memory, Plan/Organize, Organization of Materials, Monitor) and 2 validity scales (Inconsistency and Negativity). These scales form 2 global indexes (Behavioral Regulation, Metacognition, Global Executive Composite score) representing overall EF.

The Conners’ Continous Performance Test – third edition (CPT-3) is a computerized test wherein the participants are instructed to push the spacebar as soon as a target appears on the screen (a letter), except when the letter is X.^[19]^ The test is characterized by high signal-to-noise ratio by including 90% target stimuli and 10% no-target stimuli (x). The test incudes 360 trials presented across 6 blocks, with 3 subgroups with different interstimulus intervals. The test durance is 14 minutes, and the results generate measures of reaction time, accuracy, variability, consistency, and vigilance. BRIEF and CPT-3 will be used as a measure of attention and EF in patients with IBS, as a predictor of treatment response (baseline); and as an outcome variable (change from baseline to follow-up).

The NEO-FFI-3 is a 60-item version of the NEO-PI-3 providing a quick and reliable measure of the Five-Factor model of personality (Neuroticism, Extraversion, Openness, Agreeableness, and Conscientiousness).^[20,21]^ The instrument is well validated for use in several countries. Measures will include the 5 factors as well as profiles of factors for individual adults. In the present study, the personality assessment will be part of the phenotyping of adults with IBS and controls; one of the predictors of treatment response, a more general predictor of quality of life, pain, and other self-reported challenges associated with IBS, and related to the BGM axis.

#### 3.5.3 Gastric motility and ultrasonography

Gastric motility will be characterized using transabdominal ultrasound and the meal accommodation test (UMAT). The UMAT involves consumption of a standardized 500 mL, pre-boiled, low-caloric soup after which transabdominal ultrasound allows visualization and measurement of the proximal and distal compartments of the stomach.^[22–24]^ We will characterize the patient's gastric accommodation, emptying, and quantify antral contractions. Antral biopsies will be investigated for density of interstitial cells of Cajal, correlated to functional observations during the UMAT.

#### 3.5.4 Vagal activity

Pulse oximetry data will be acquired during the resting-state fMRI sequence in order to collect (heart) interbeat intervals (IBIs), with a photoplethysmograph placed on the right index finger (50 Hz). Pulse oximetry data offers an especially accurate approximation of interbeat intervals and is associated with less artifacts in a MR-environment. In order to control for the effect of respiration in the pulse oximetry data, a chest strain gauge will be applied to measure respiratory frequency. Heart rate variability (HRV) and breathing pattern is registered during resting state, that is, the time the patient is in the MR machine in supine position with spontaneous breathing (approximately 20 minutes). The pulse oximetry data will be used to calculate HRV. In order to estimate the parasympathetic modulation for heart rate, an estimate of fast IBI changes in the time scale of milliseconds will be obtained, that is, faster changes that sympathetic modulation for heart rate. Thus, high frequency HRV > 0.14 Hz and time-domain measures reflecting such fast changes ([-mean-square of successive R-R-interval differences (RMSSD)] provide a readily available, estimate of vagal activity.^[25]^

#### 3.5.5 Assessment of low-FODMAP dietary response

A subgroup of patients with IBS-D (n = 60) will undergo a strict 12-week low-FODMAP dietary intervention guided by clinical dietitian. Here, patients will exclude FODMAPs from their diet without reintroduction during the entire study period. Patients will meet with their registered dietitian four times (baseline, week 4, 8, and 12). At baseline, the patients will be informed about the diet intervention and the low-FODMAP-diet. They will fill out questionnaires and records of the diet (3 days) are collected at week 2, 4, 6, 8, 10, and 12 (Table 1). The registered dietitian is available for the patients by phone and e-mail throughout the entire study period.

#### 3.5.6 Biological tissue sampling

Routine gastroscopy and sigmoidoscopy will be carried out to exclude other pathologies, and biopsies will be harvested from the antrum, duodenum, and sigmoid colon for histological and molecular biomarker investigation. Saliva will be sampled for oral microbiota composition. Blood samples (serum, plasma, buffy coat) will be collected for analysis of GI hormones, serotonin levels, gut integrity markers, and pro-inflammatory cytokines and interleukins). Stool samples will be collected for gut microbiota profiling and estimates of abundance and functions.

#### 3.5.7 Questionnaires

During baseline and multiple time-points throughout the dietary intervention (Table 1), standardized questionnaires on intestinal and extraintestinal symptomatology, sleep, fatigue, and quality of life will be assessed: Irritable Bowel Syndrome Quality of Life Measure (IBS-QOL),^[26]^ Irritable Bowel Syndrome – Symptom Severity Score (IBS-SSS),^[8]^ Hospital Anxiety and Depression scale (HADS),^[27]^ Bristol Stool Scale (BSS),^[28]^ Visceral sensitivity index (VSI),^[29]^ Gastrointestinal Symptom Rating Scale for Irritable Bowel Syndrome (GSRS-IBS),^[30]^ IBS symptom questionnaire (IBS-SQ),^[31,32]^ Chalder fatigue scale (CFQ-11),^[33]^ Wender Utah Rating Scale for ADHD in adults (WURS),^[34]^ Autism-spectrum quotient (AQ),^[35]^ and Bergen Insomnia Scale (BIS),^[36]^ Hospital Anxiety and Depression scale (HADS),^[27]^ Bristol Stool Scale (BSS),^[28]^ Visceral sensitivity index (VSI),^[29]^ Gastrointestinal Symptom Rating Scale for Irritable Bowel Syndrome (GSRS-IBS),^[30]^ IBS symptom questionnaire (IBS-SQ),^[31,32]^ Chalder fatigue scale (CFQ-11),^[33]^ Adult ADHD self-report scale (ASRS),^[37]^ Wender Utah Rating Scale for ADHD in adults (WURS),^[34]^ Autism-spectrum quotient (AQ),^[35]^ and Bergen Insomnia Scale (BIS).^[36]^

#### 3.5.8 Analysis of microbiota composition, short-chain fatty acids, and shotgun metagenomics

The DNA will be extracted from stool samples using the QIAamp Fast DNA Stool Mini Kit (Qiagen, Hilden, Germany), according to the manufacturer's instructions, with a prior step of bead beating in 2 mL microcentrifuge tubes containing 0.1 mm diameter glass beads, ∼ 150 mg faeces, and 1 mL InhibitEX buffer (Qiagen, Hilden, Germany). The faecal DNA concentration will be measured by fluorescence-based methods such as Qubit 3.0 and the Qubit dsDNA HS Assay Kit (Thermo Fisher Scientific, Waltham, MA). Library preparation for shotgun metagenomic sequencing will be performed using the Nextera^TM^ DNA Flex Library Preparation kit (Illumina, San Diego, CA) from 350 to 450 ng of gDNA. A low cycling indexing protocol (5 PCR cycles) and Unique Dual Indices will be used to minimize amplification bias and to prevent index hopping respectively. Every final library will be individually quantified on the Qubit 3.0 fluorometer and run on a Bioanalyzer HS DNA chip (Agilent, Santa Clara, CA) to verify quality and size distribution. The 2 x 150nt paired-end sequencing will be conducted on a NovaSeq 6000 sequencer (Illumina, San Diego, CA, USA). All samples will be accommodated in one S4 flow cell to minimize the bias of batch sequencing. Paired-end fastq files will be used to perform a phylogenetic marker gene-based operational taxonomic units (mOTUs) analyses, enabling the taxonomy profiling of >7700 microbial species.^[38]^ In addition, reconstruction of metagenome assembled genomes (MAGs) will permit assess gut microbiota functionality with different representation across the intervention time points.

Feces will be analysed for short-chain fatty acids (SCFA). About 0.5 g of the fecal material will be homogenised after addition of distilled water containing 3 mmol/L of 2-ethylbutyric acid (as internal standard) and 0.5 mmol/L of H_2_SO_4_; 2.5 mL of the homogenate will be vacuum distilled, according to the method of Zijlstra et al, as modified by Høverstad et al.^[39]^ The distillate will be analyzed with gas chromatography (Agilent 7890 A, CA), using a capillary column (serial no. USE400345H; Agilent J&W GC columns, CA), and quantified using internal standardization. Flame ionisation detection will be employed. In addition, we will calculate the proportional distribution of individual SCFA to total SCFA.

Analysis of salivary microbiota composition will be performed using PCR. A total of 5 mL unstimulated saliva is collected from each patient and healthy control using a 50 mL sterile Falcon tubes. Each sample is then immediately placed in an ice bag and stored at –80°C until extractions. DNA from the collected saliva samples will be extracted using a TIANamp Bacteria DNA Kit (Tiangen Biotech, Beijing, China) following the manufacturer's instructions. Genomic DNA will be used as the template for bacterial 16S rRNA gene amplification with the barcoded primers 338F (50-ACTCCTACGGGAGGCAGCAG-30) and 806R (50- GGACTACHVGGGTWTCTAAT-30), which target the V3–V4 hypervariable region. A real-time PCR system combination with the Illumina MiSeq platform (16S rRNA) (Illumina, San Diego, CA) will be used for sequencing of oral microbiome profile.^[40]^

#### 3.5.9 Data management and machine learning

Data will be collected during consultations at Haukeland University Hospital (HUH). Specific patient-reported outcome data (questionnaires: HADS, Rome-III, IBS-SSS) are collected electronically using CheckWare (https://checkware.com), security level 4 (e.g., with BANK-ID). All patient data will be plotted into an electronic database, FileMakerPro 17 Advanced, stored at Haukeland University Hospital Research server (2015–01621). All data are registered using the patient's study-ID, and the study participant-identifying key is stored in a separate location at the research server. No patient data are located in proximity to the study participant-identifying key. Biological material such as GI biopsies, blood (serum, plasma, etc), stool samples, and saliva is collected in 2D barcode labeled tubes (Matrix), scanned, and stored in Biobank #2176 at Haukeland University Hospital.

**Figure 1:**
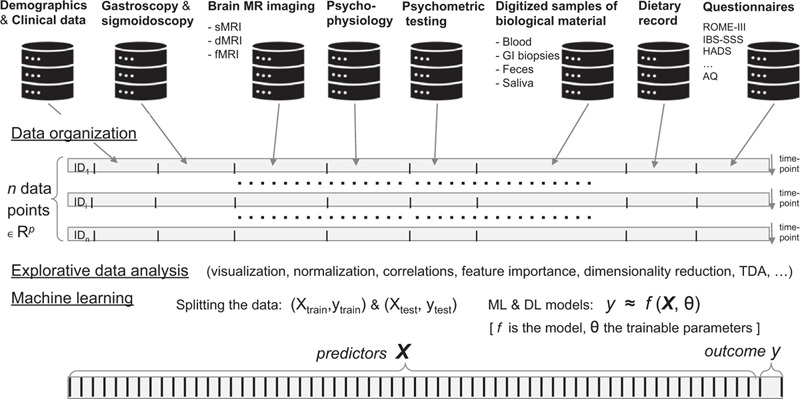
The brain-gut-microbiota spatiotemporal digital biobank. Patients with IBS will be using the same CRF, assessing extrinsic factors, medications, and family history. Blood samples, stool, saliva, and gastrointestinal tissue will be sampled. Established, validated questionnaires to assess GI symptoms, comorbid conditions, and somatization will be collected. Data from multimodal brain imaging examination, detailed measurements of GI function, and psychometric information from tests and questionnaires, dietary records, microbiota-profiles, pain-provocation tests results, and dietary responsiveness will be fed into a spatiotemporal digital biobank for biostatistics integration of data and modelling to identify novel subgroups for personalized treatment.

In this large and heterogeneous data set collected from multiple sources from the individual participant in the study, we will employ exploratory data analysis (EDA) to get insight, and state-of-the-art supervised and unsupervised machine learning techniques for predictions, and detection of IBS subgroups and identification of diagnostic biomarkers (Fig. 1). More specifically, we will use Python (https://www.python.org) and Jupyter notebooks (https://jupyter.org) to support open science and reproducible research.^[41,42]^ For implementing machine learning procedures and pipelines, we will use Pandas Numpy, Scikit-learn (https://scikit-lern.org). For deep learning approaches, especially related to the MRI acquisitions, we will consider the Pytorch (https://pytorch.org) framework and the high-performing fastai (https://docs.fast.ai) libraries using deep learning best practices allowing models to train fast with limited resources. We will address the importance of separate training, validation and test datasets, and employ cross-validation techniques to reduce overfitting and improve generalization to any data from the problem domain. Before employing machine learning techniques, for processing the acquired neuroimaging data, we will consider using Freesurfer (http://surfer.nmr.mgh.harvard.edu), FSL (https://fsl.fmrib.ox.ac.uk/fsl/fslwiki/), and AFNI (https://afni.nimh.nih.gov/).^[43]^ Freesurfer will mainly be used for cortical reconstruction and volumetric segmentation to extract the region of interest (ROI). We have planned to used FSL and AFNI to obtain diffusion and functional maps, respectively.

## Acknowledgments

We would like to thank all the staff and patients involved with the study at the Department of Medicine, Section for Gastroenterology, Department of Radiology and the Center for Nuclear Medicine and PET, Department of Pathology, Mohn Medical Imaging and Visualisation Center, at Haukeland University Hospital for their ongoing support.

## Author contributions

All authors made a significant contribution to the conception and design of the protocol. BB made major contributions to the design of this study, ethical approval application, development of the protocol, and drafting the manuscript. GAL, EKS, BB, TH, EPT, ERH designed the study and dietary intervention, EKS, OHG, TH designed the UMAT test, BO designed the protocol for vagal activity measures, GAL and IB designed the biochemistry analysis plan, YA, ABP, JV designed the protocol and plan for microbiota analysis, EAM, AG and AL developed and designed the advanced neuroimaging protocol, AL and BHN developed the big data statistical analysis and machine-learning plan. AJL designed the protocol for all psychometric tests. AL, TH, OHG, JGH, GAL, and BB secured funding. All authors contributed to refinement of the study protocol and the final manuscript.
